# Development of a high-throughput screen to identify small molecule enhancers of sarcospan for the treatment of Duchenne muscular dystrophy

**DOI:** 10.1186/s13395-019-0218-x

**Published:** 2019-12-12

**Authors:** Cynthia Shu, Ariana N. Kaxon-Rupp, Judd R. Collado, Robert Damoiseaux, Rachelle H. Crosbie

**Affiliations:** 1 Molecular Biology Institute, University of California Los Angeles, Los Angeles, USA; 2 Department of Integrative Biology and Physiology, University of California Los Angeles, 610 Charles E. Young Drive East, Terasaki Life Sciences Building, Los Angeles, CA 90095 USA; 3 Center for Duchenne Muscular Dystrophy, University of California Los Angeles, Los Angeles, USA; 4 Department of Molecular and Medicinal Pharmacology, University of California Los Angeles, Los Angeles, USA; 5 California NanoSystems Institute, University of California Los Angeles, Los Angeles, CA USA; 6 Department of Neurology David Geffen School of Medicine, University of California Los Angeles, 610 Charles E. Young Drive East, Terasaki Life Sciences Building, Los Angeles, CA 90095 USA

**Keywords:** Drug discovery, Duchenne muscular dystrophy, C2C12, Dystrophin, Felodipine, High-throughput screen, Sarcolemma, Sarcospan, Small molecules

## Abstract

**Background:**

Duchenne muscular dystrophy (DMD) is caused by loss of sarcolemma connection to the extracellular matrix. Transgenic overexpression of the transmembrane protein sarcospan (SSPN) in the DMD *mdx* mouse model significantly reduces disease pathology by restoring membrane adhesion. Identifying SSPN-based therapies has the potential to benefit patients with DMD and other forms of muscular dystrophies caused by deficits in muscle cell adhesion.

**Methods:**

Standard cloning methods were used to generate C2C12 myoblasts stably transfected with a fluorescence reporter for human SSPN promoter activity. Assay development and screening were performed in a core facility using liquid handlers and imaging systems specialized for use with a 384-well microplate format. Drug-treated cells were analyzed for target gene expression using quantitative PCR and target protein expression using immunoblotting.

**Results:**

We investigated the gene expression profiles of SSPN and its associated proteins during myoblast differentiation into myotubes, revealing an increase in expression after 3 days of differentiation. We created C2C12 muscle cells expressing an EGFP reporter for SSPN promoter activity and observed a comparable increase in reporter levels during differentiation. Assay conditions for high-throughput screening were optimized for a 384-well microplate format and a high-content imager for the visualization of reporter levels. We conducted a screen of 3200 compounds and identified seven hits, which include an overrepresentation of L-type calcium channel antagonists, suggesting that SSPN gene activity is sensitive to calcium. Further validation of a select hit revealed that the calcium channel inhibitor felodipine increased SSPN transcript and protein levels in both wild-type and dystrophin-deficient myotubes, without increasing differentiation.

**Conclusions:**

We developed a stable muscle cell line containing the promoter region of the human SSPN protein fused to a fluorescent reporter. Using the reporter cells, we created and validated a scalable, cell-based assay that is able to identify compounds that increase SSPN promoter reporter, transcript, and protein levels in wild-type and dystrophin-deficient muscle cells.

## Background

Duchenne muscular dystrophy (DMD) is a progressive muscle wasting disorder that affects approximately 1 in every 5700 males, making it the most common lethal genetic disorder in children [1]. Individuals with DMD lack dystrophin protein, which normally stabilizes myofibers by connecting the actin cytoskeleton, through the sarcolemma, to the extracellular matrix (ECM) [2, 3]. Loss of this essential connection leads to contraction-induced damage of the cell membrane and myofiber [4]. Dystrophin-deficient muscles undergo asynchronous cycles of degeneration and satellite cell-mediated regeneration (for review, [5]). As the population of satellite cells is diminished, the regenerative capacity is reduced and muscle undergoes end-stage pathology, characterized by the replacement of myofibers with fibrotic and adipose tissue [5]. Patients lose the ability to walk at approximately 10 years of age, but with corticosteroid treatment, the initial age of wheelchair reliance is extended to 13 years of age [6]. Approximately 90% of patients over 18 years of age develop dilated cardiomyopathy and respiratory dysfunction, leading to premature death in the third decade of life [1, 7].

Mutations in the dystrophin gene lead to loss of dystrophin protein from the sarcolemma resulting in DMD [2]. Dystrophin localizes to the cytoplasmic side of the sarcolemma in myofibers and cardiomyocytes, where it binds to the actin cytoskeleton and β-dystroglycan [8]. Dystrophin interacts with transmembrane and peripheral membrane proteins (dystroglycan, sarcoglycans, and sarcospan) to form the dystrophin-glycoprotein complex (DGC), which serves as a receptor for laminin the ECM [9–13]. The physical axis of connection from actin to the ECM is crucial for maintaining membrane integrity. Loss of dystrophin leads to an absence of the entire DGC at the sarcolemma, leaving the myofiber vulnerable to contraction-induced damage [14].

In addition to the DGC, skeletal muscle contains two other major adhesion complexes, the utrophin-glycoprotein complex (UGC) and the α7β1D-integrin complex, that also interact with actin inside the myofiber and bind to laminin and other ligands in the ECM [15, 16]. The composition of the UGC is highly similar to that of the DGC with the main exception that utrophin, a 395 kDa autosomal paralogue of dystrophin, mediates the interaction between β-dystroglycan and the actin cytoskeleton [17]. The UGC localizes to the neuromuscular and myotendinous junctions in healthy skeletal muscle and throughout the sarcolemma in fetal and regenerating muscle [17–19]. Utrophin overexpression studies in the genetic mouse model of DMD (*mdx*) demonstrated that a three- to fourfold increase of the UGC at the sarcolemma compensated for dystrophin deficiency [20, 21]. The α7β1D-integrin complex connects the sarcolemma to laminin and was also able to significantly rescue disease pathology when overexpressed in *mdx* mice [22, 23].

Sarcospan (SSPN) is a 25 kDa transmembrane protein expressed in skeletal and cardiac muscle and interacts with all three adhesion complexes [13, 24, 25]. SSPN tightly associates with the sarcoglycan subcomplex that is associated with dystrophin and utrophin [24, 26, 27]. Transgenic overexpression of SSPN in dystrophin-deficient *mdx* mice (*mdx:*SSPN-Tg) stabilized the sarcolemma by increasing the abundance of the UGC and α7β1D-integrin complex at the membrane, effectively restoring laminin binding [25, 27–29]. SSPN overexpression in *mdx* mice improved membrane integrity, revealed by reduced membrane-impermeable dye uptake and decreased serum levels of muscle creatine kinase [30]. SSPN-mediated strengthening of the sarcolemma improved resistance to degeneration, indicated by a decrease in central nucleation, a marker of myofiber turnover [30]. These improvements at the cellular level translated to functional improvements in post-exercise activity levels and eccentric contraction-induced force drop assays [30]. SSPN overexpression also addressed cardiac and pulmonary complications, which are the leading causes of death in DMD patients. *Mdx*:SSPN-Tg mice exhibited an improvement in cardiomyocyte membrane integrity, enhanced cardiac function, and increased adhesion complex localization at the cardiomyocyte membrane [31]. The transgenic mice showed improved minute ventilation and peak expiratory flow, indicators of pulmonary fitness [30]. Expression of SSPN-Tg in skeletal muscles of utrophin-null and α7-integrin-null mice on a *mdx* background confirmed that the rescue effect of SSPN is dependent on the presence of both the UGC and the α7β1D-integrin complex [28, 29].

Knockout studies provide further insight into the mechanism of SSPN as a therapy and in the context of disease. While SSPN-null mice lack an obvious muscle phenotype at baseline, they exhibited reduced membrane levels of the DGC and UGC and increased levels of the α7β1D-integrin complex [25, 32]. SSPN-deficient skeletal muscle showed reduced laminin binding and increased susceptibility to eccentric contraction-induced damage at older ages. Cardiotoxin injury of the SSPN-null muscle revealed a diminished regenerative capacity, reduced activation of the pro-regenerative Akt/p70s6K signaling pathway, and reduced regeneration-induced utrophin upregulation response [28]. This diminished regenerative response places SSPN as an upstream regulator of the Akt pathway, a regulator of myofiber repair. In the heart, loss of SSPN exacerbated cardiac hypertrophy and fibrosis after isoproterenol-induced β-adrenergic challenge, revealing the protective role of SSPN in the context of cardiac stress and disease [31]. Overall, these studies concluded that SSPN is a regulator of adhesion complex localization and provides stability to the sarcolemma in both skeletal and cardiac muscle (for review, [33, 34]).

The development of therapies for DMD is gaining momentum with the accelerated approval of eteplirsen in 2016 and the increased private sector funding of rare disease programs. However, the existing FDA-approved drugs for DMD are not sufficient to substantially slow disease progression. While corticosteroids dampen inflammation and extend ambulation by several years, they do not address adhesion complex and membrane stability deficiencies. In clinical trials, the antisense oligonucleotide exon skipping therapy eteplirsen increased truncated dystrophin protein production, but at a controversially low rate [35]. Our preclinical studies in *mdx* mice support SSPN as a stand-alone or combinatorial therapeutic target for DMD and Becker muscular dystrophy because of its ability to facilitate localization and stability of compensatory adhesion complexes. Importantly, SSPN overexpression to 30-fold levels does not lead to detrimental side effects in mice, making it a favorable candidate to pursue as a therapy [30]. Small molecule therapies, which comprise over 80% of all approved drugs, are especially appealing due to their ability to bypass the limitations of delivery, repeat dosages, and immune responses seen with virus-mediated gene delivery and stem cell–based approaches [36]. In the case of rare diseases such as DMD, the ability to bring patient-specific gene correction therapies to clinic is not yet feasible. SSPN is expected to benefit all cases of DMD regardless of genetic mutation. In this study, we report the development of a cell-based high-throughput assay to identify small molecule enhancers of SSPN gene expression for the treatment of DMD.

## Methods

### Gene expression analysis

RNA from myotubes treated for 48 h was extracted from cells using Trizol-based (Thermo Fisher Scientific) phase separation, as previously described [37]. RNA concentrations were determined using a NanoDrop 1000 (Thermo Fisher Scientific), and 750 ng of RNA in a 20-μl reaction was reverse transcribed using iScript cDNA synthesis (Bio-Rad) with the following cycling conditions: 25 °C for 5 min, 42 °C for 30 min, 85 °C for 5 min. For quantitative PCR, SsoFast EvaGreen Supermix (Bio-Rad), 400 nM of each optimized forward and reverse primer (for primer descriptions see Additional file 1: Table S1), and cDNA corresponding to 37.5 ng RNA were used to amplify cDNA measured by Applied Biosystems 7300 (Thermo Fisher Scientific) with the following reaction conditions: 55 °C for 2 min, 95 °C for 2 min, 40 cycles of 95 °C for 10 s and 62 °C for 30 s, and dissociation stage. Each sample was run in triplicate. Data was analyzed using the ddCT method and normalized to reference gene, GAPDH, or β-actin, with vehicle-treated samples serving as the calibrator (relative expression of vehicle control = 1).

### Molecular cloning of SSPN reporter plasmids

The SSPN promoter region was predicted using publicly available data on the UCSC Genome Browser (http://genome.ucsc.edu/). Using the GRCh37/hg19 assembly, gene regulatory elements of the cardiac and skeletal muscle-specific SSPN transcript variant 1 (NM_005086.4) of the human SSPN gene (NG_012011.2) were identified. H2K4me3 marks, DNase hypersensitivity regions, and ChIP-seq data showing transcription factor binding locations from human skeletal muscle cultures indicated the promoter region to be upstream of exon 1 and within exon 1 (Additional file 2: Figure S1). A 2-kb region encompassing the human SSPN promoter was amplified from human genomic DNA (Bioline) using Phusion High Fidelity DNA Polymerase (New England Biolabs) with the primers indicated in Additional file 3: Table S2. The primers contained leader sequences and restriction sites for BglII (AGATCT) or HindIII (TTCGAA). The PCR products were purified using the PureLink HiPure Plasmid DNA Purification kit (Life Technologies) and digested with BglII and HindIII in NEBuffer 3.1 (New England Biolabs). The 2-kb digested PCR products were electrophoresed on agarose gels, excised, and purified using the Zymoclean Gel DNA Recovery Kit (Zymo Research). pmEGFP-1 (Addgene, plasmid #36409) was digested with BglII and HindIII and ligated to PCR products using T4 DNA ligase (Invitrogen). The plasmid constructs were linearized with BglII, which digested the region upstream of the SSPN promoter. The linearized plasmids were purified and used to transform One Shot TOP10 chemically competent *E. coli* (Thermo Fisher) grown on agar containing the appropriate antibiotic. Individual colonies were confirmed by colony PCR to contain the SSPN promoter construct and inoculated in liquid culture overnight. The plasmids were purified using PureLink Quick Plasmid Miniprep (Life Technologies) and subjected to DNA sequencing (Laragen Inc.) using the primers in Additional file 3: Table S2 to confirm presence and accuracy of the SSPN promoter region. Select bacterial cultures were grown in large cultures and collected for plasmid purification using the Plasmid Maxi Kit (Qiagen).

### Generation of stable reporter cell lines

C2C12 immortalized murine myoblasts cultured in growth media consisting of DMEM (Gibco) with 20% fetal bovine serum (Sigma-Aldrich) at 37 °C with 5% CO_2_ were transfected with the hSSPN-EGFP linearized plasmid using Lipofectamine 3000 Transfection Reagent (Thermo Fisher Scientific). Transfected cells were selected using 800 μg/ml of G418 (Sigma-Aldrich) for 4 weeks to generate stable cell lines expressing reporter protein under control of the human SSPN promoter.

### High-throughput screening

hSSPN-EGFP myoblasts were seeded at 500 cells per well in 50 μl of growth media in 384-well black, clear bottom microplates (Greiner) using a Multidrop 384 (Thermo Fisher Scientific) and incubated for 3 days. Upon reaching confluency, the growth media was replaced with 50 μl of differentiation media consisting of DMEM with 2% horse serum (Sigma-Aldrich) using an EL406 combination washer dispenser (Biotek). At day 2 of differentiation, the media on the cells were aspirated, left with a residual volume of 10 μl, and replaced with 30 μl of fresh differentiation media. 0.5 μl of small molecule in DMSO or DMSO alone (for vehicle and positive control wells) was added to each well using a Biomek Fx (Beckman). To ensure proper mixing of the DMSO, 50 μl of additional differentiation media was added to all wells except the positive control treated wells, which instead received 50 μl of media containing insulin transferrin selenium (ITS) (Gibco) to reach a final concentration of 1% ITS. The final concentration of drug in each treated well was 5.5 μM in 0.55% DMSO and 0.55% DMSO only for vehicle and positive control treated wells. After 48 h of incubation, the media were replaced with Fluorobrite DMEM (Gibco) and each plate was imaged using ImageXpress Micro Confocal High Content Imaging System (Molecular Devices). The fluorescence intensity of imaged cells was determined using a custom module analysis in MetaXpress Analysis software (Molecular Devices). Analysis setting were as follows: top hat (size, 12; filter shape: circle), adaptive threshold (source: top hat; minimum width, 10; maximum width, 800; intensity above local background, 500), filter mask (filter type: minimum area filter; minimum value, 500).

### Cell culture

C2C12 cells (American Type Culture Collection) were grown at 37 °C with 5% CO_2_ in growth media containing DMEM (Gibco) with 20% FBS (Sigma-Aldrich). Upon reaching 90–100% confluency, the media was replaced with differentiation media containing DMEM with 2% horse serum (Sigma-Aldrich). Conditionally immortalized H2K *mdx* myoblasts [38] with a nonsense mutation in exon 23 of dystrophin were a gift from Terrance Partridge, Ph.D. (Children’s National Medical Center, Washington, D.C.) [38]. Cells were allowed to proliferate on 0.01% gelatin (Sigma-Aldrich) coated plates at 33 °C with 5% CO_2_ with growth media containing DMEM, 20% HI-FBS (Invitrogen), 2% L-glutamine (Sigma-Aldrich), 2% chicken embryo extract (Accurate Chemical), 1% penicillin-streptomycin (Sigma-Aldrich), and 20 U/ml of fresh interferon gamma (Gibco). For differentiation, H2K *mdx* myoblasts were seeded on plates coated with 0.1 mg/ml matrigel (Corning) diluted in DMEM and grown in proliferation conditions. Upon reaching 90–100% confluency, cells were grown at 37 °C with 5% CO_2_ in differentiation media containing DMEM with 5% horse serum (Sigma-Aldrich), 2% L-glutamine, and 1% penicillin-streptomycin using established protocols [38]. RAW264.7 macrophages (ATCC), a gift from Amy Rowat, Ph.D. (University of California, Los Angeles), were cultured in DMEM containing 20% FBS at 37 °C with 5% CO_2_.

### In vitro treatments

Cells were treated for 48 h beginning at day 2 of differentiation with DMSO (vehicle control, ATCC), aceclidine (Sigma-Aldrich), acyclovir (Sigma-Aldrich), alloxazine (Sigma-Aldrich), carbadox (Sigma-Aldrich), felodipine (Sigma-Aldrich), GW5074 (Sigma-Aldrich), isoproterenol (Sigma-Aldrich), isradipine (Sigma-Aldrich), lacidipine (Sigma-Aldrich), nafadotride (Tocris Bioscience), nandrolone (Sigma-Aldrich), nifedipine (Sigma-Aldrich), nilvadipine (Sigma-Aldrich), alloxazine (Sigma-Aldrich) diluted in cell-type specific differentiation media at doses listed in figures.

### Immunoblotting

C2C12 murine myotubes treated for 48 h were lysed using RIPA buffer (Thermo Fisher Scientific) containing a protease inhibitor cocktail (0.6 μg/ml pepstatin A, 0.5 μg/ml aprotinin, 0.5 μg/ml leupeptin, 0.1 mM PMSF, 0.75 mM benzamidine, 5 μM calpain I inhibitor, 5 μM calpeptin). Cell lysates in RIPA buffer were rocked for 1 h at 4 °C and centrifuged at 1000 RPM for 30 min at 4 °C. The supernatant was collected, quantified for protein concentration using the DC protein assay (Bio-Rad), and normalized to 2 mg/ml in water and Laemmli sample buffer with a final concentration of 10% glycerol (Sigma-Aldrich), 5% beta-mercaptoethanol (Sigma-Aldrich), 3% sodium dodecyl sulfate (Sigma-Aldrich), and 0.05% bromophenol blue (Sigma-Aldrich). For SDS-PAGE, samples were heated to 95 °C for 2 min before loading 40 μg to a 4–12% Tris-glycine gel (Novex), run for 2 h at 100 V at RT, and transferred to a nitrocellulose membrane for 2 h at 100 V at 4 °C. Ponceau S staining was performed to visualize protein loading. Membranes were blocked with 5% blotto (5% non-fat dried milk) in Tris-buffered saline with 0.1% tween-20 (TBST, Sigma-Aldrich) for 1 h at RT and incubated on a rocker overnight at 4 °C with the following primary antibodies and dilutions in 5% blotto: SSPN (sc-393,187, 1:200, Santa Cruz Biotechnology), GAPDH (Mab374, 1:10,000, Millipore). Following three 10-min TBST washes, the membranes were incubated in goat anti-mouse IgG HRP (Ab6789, 1:5000 for SSPN, 1:10,000 for GAPDH, Abcam) diluted in 5% milk for 2 h at RT. The membranes were then washed three times for 10 min each with TBST, incubated in SuperSignal West Pico Chemiluminescent Substrate (Thermo Fisher Scientific) for 5 min at RT on an orbital shaker, and exposed to autoradiography films (Agfa). Autoradiography films were developed using a SRX-101A tabletop processor (Konica Minolta), scanned to a digital file, and analyzed by densitometry of bands using ImageJ version 1.51 s [39]. Target protein bands were normalized to loading control GAPDH with vehicle-treated cells serving as the calibrator sample (relative protein levels of vehicle control = 1).

### Fusion index

Cells in a 96-well plate treated for 72 h beginning at day 2 of differentiation were fixed with 4% PFA for 20 min, permeabilized with 0.2% Triton X-100 (Sigma) for 10 min, and blocked with 1% BSA for 30 min. Myosin heavy chain (MHC) was detected using 10 μg/ml MF-20 (Developmental Hybridoma Studies Bank) in 1% BSA overnight and 10 μg/ml goat anti-mouse Alexa Fluor Plus 594 (Thermo Fisher Scientific) in 1% BSA for 1 h. PBS washes were performed between each step above. Nuclei were stained with 5 μg/ml Hoechst (Thermo Fisher Scientific) for 20 min before imaging. Each treatment was performed in three wells and three fields per well were captured. ImageJ was used to count the number of total nuclei and nuclei within a MHC-positive cell. Fusion index was calculated as nuclei in MHC-positive cell/total nuclei.

### Cell viability assay

Cells in a 384-well plate were treated for 48 h beginning at day 2 of differentiation. CellTiter-Glo Luminescent Cell Viability Assay was used to assess cell viability by adding the assay reagent, and media on cells at a ratio of 1:1. Luminescence was measured using a standard plate reader.

### Statistics

Robust strictly standardized mean difference (SSMD*) was used to assess plate quality and for hit selection. SSMD* = *X*_P_ – *X*_N_/1.4826 $\sqrt{\ {s}_P^2+{s}_N^2\ }$, where *X*_P_, *X*_N_, *S*_P_, and *S*_N_ are the medians and median absolute deviations of the positive and negative controls, respectively [40]. For plate quality, SSMD* ≥ 1 indicates a good quality moderate positive control. For initial hit selection, a 1.4-fold increase over vehicle and SSMD* > 0.25 was considered a hit. Statistical analysis was performed using Prism version 7.0 (GraphPad Software) for Mac OS X using the two-tailed, non-parametric Kolmogorov-Smirnov test. Data are reported as mean ± SEM. A *p* value of < 0.05 was considered statistically significant. *p < 0.05; **p < 0.01; ***p < 0.001; ****p < 0.0001.

## Results

Using transgenic overexpression of SSPN in *mdx* mice, we have previously established that as little as threefold induction of SSPN expression ameliorates the cardiac, respiratory, and skeletal muscle symptoms in murine models of DMD [33]. To identify a translational SSPN-based therapy, we evaluated in vitro model systems, reporters, and assay conditions to develop a high throughput assay for SSPN enhancers that can be used in 384-well format to efficiently survey large chemical libraries.

### Defining model systems for screening by evaluating target gene expression during myotube differentiation

**Fig. 1:**
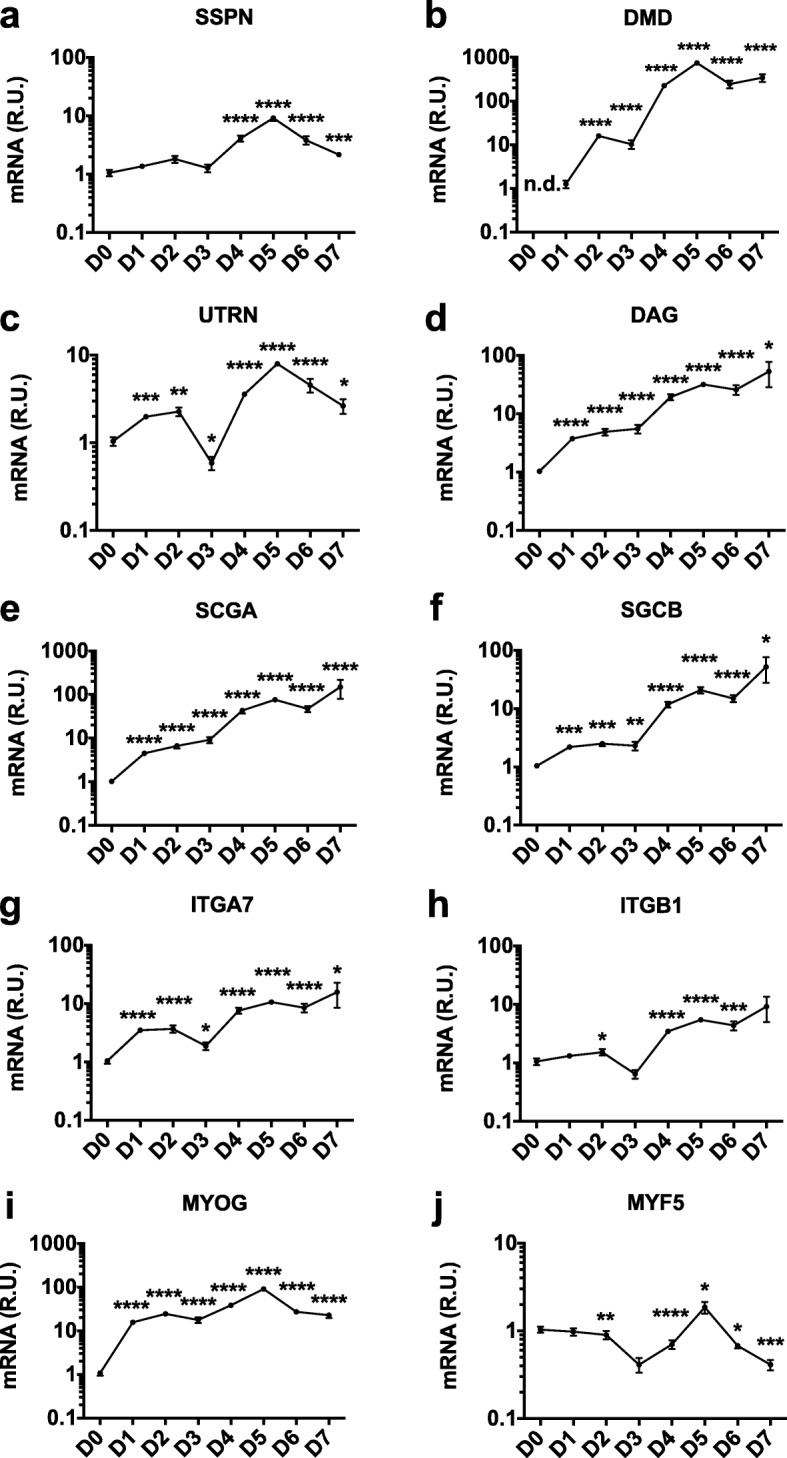
Individual components of muscle adhesion complexes increase during C2C12 differentiation. Confluent C2C12 myoblasts (day 0, D0) were switched from proliferation to differentiation media and harvested daily for 7 days (D1 to D7). Individual genes encoding protein components of the three major adhesion complexes (DGC, UGC, and α7β1D-integrin complex) were investigated, including **a** SSPN, sarcospan; **b** DMD, dystrophin; **c** UTRN, utrophin; **d** DAG, dystroglycan; **e** SCGA, α-sarcoglycan; **f** SCGB, β-sarcoglycan; **g** ITGA7, α7 integrin; and **h** ITGB1, β1D integrin. Analysis of myogenin (MYOG, **i**) and myogenic factor 5 (MYF5, **j**) are provided as markers for muscle cell differentiation. For DMD D0, n.d. (no data) indicates no detectable expression. Gene expression was calculated using the ddCt method and normalized to β-actin with day 0 (myoblast) values serving as the calibrator sample (*n* = 3). R.U., relative units. Results are plotted on a log scale. *p < 0.05, **p < 0.01, ***p < 0.001, ****p < 0.0001

As a first step to identifying enhancers of expression, we sought to determine an appropriate cell-based model system. Although SSPN is expressed in a wide range of tissues, we wanted to focus the assay on screening in the context of skeletal muscle and, for this reason, selected immortalized C2C12 myoblasts for the screen due to their ease of culture and ability to grow in large quantities. However, it was unclear to us whether to conduct the assay using immature myoblasts or mature, differentiated myotubes. In order to understand SSPN gene activity in myoblasts and myotubes, we directly interrogated SSPN gene expression in C2C12 cells at each day during differentiation for a total of 7 days. Cells undergo fusion starting on day 3 and appear to be fully differentiated at day 6, as previously reported [41]. We found that SSPN mRNA abundance is relatively unchanged during the first 3 days following the incubation of C2C12 cells in differentiation media (Fig. 1a). However, SSPN levels begin to increase exponentially at day 3, reaching tenfold levels by day 5. The increased SSPN gene expression occurs just after the first evidence of visible myotube fusion at day 3 (Additional file 4: Figure S2). We also evaluated the expression of SSPN associated proteins including dystrophin (DMD), utrophin (UTRN), dystroglycan (DAG), α7 integrin (ITGA7), β1D integrin (ITGB1), α-sarcoglycan (SGCA), and β-sarcoglycan (SGCB). We found that gene activity increased immediately after myoblasts were switched from proliferation to differentiation media (Fig. 1b–i and Additional file 5: Figure S3). While DMD transcripts were not detectable in myoblasts, mRNA levels increased exponentially during differentiation. UTRN, ITGA7, and ITGB1 expression increased by tenfold, while the SCGA, SCGB, and DAG increased nearly 100-fold during the same time period (Fig. 1). As myogenic controls, we evaluated muscle-specific transcription factors, myogenin (MYOG) and myogenic factor 5 (MYF5), that are known regulators of myoblast differentiation [42]. As expected, MYOG gene activity increased immediately upon exposure to differentiation conditions (Fig. 1i), while MYF5 gradually decreased (Fig. 1j). Based on these results, we chose to treat cells at day 2 of differentiation and assay at day 4 of differentiation as this would allow for an increase in gene expression while minimizing possible saturation of gene activation.

### Generation and validation of biologically relevant reporters of SSPN gene activity

**Fig. 2:**
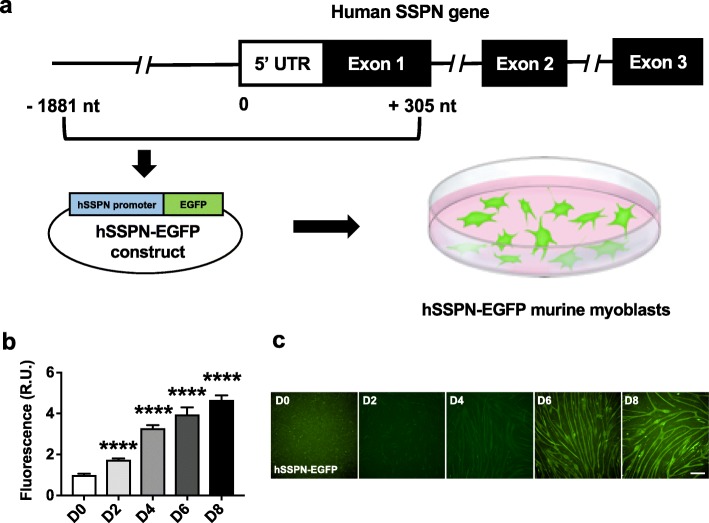
Generation and validation of biologically relevant myoblast line show effective reporting of sarcospan gene activity. The promoter region of the muscle-specific human sarcospan (hSSPN) gene was predicted using UCSC genome browser gene regulatory data. Using traditional cloning techniques, **a** A 2-kb region of the predicted hSSPN promoter was amplified and inserted into a promoterless EGFP plasmid. The construct was used to transfect C2C12 murine myoblasts reporter cell lines, which were then selected for stable transfection using antibiotic selection. Confluent myoblasts (day 0, D0) were switched from proliferation to differentiation media and assayed every other day for 8 days (D2 to D8). **b**, **c** High-content imaging of differentiating hSSPN-EGFP cells showed a transition to mature myotube morphology that correlates with an increase in reporter signal (*n* = 88 per time point). Fluorescence values are reported as relative fold change over myoblast values (R.U., relative units). Scale bar, 100 μm. ****p < 0.0001

To identify the human SSPN (hSSPN) promoter region, we used publicly available data from the UCSC Genome Browser. By assessing H2K4me3 marks, DNase hypersensitivity regions, and ChIP-seq data showing transcription factor binding sites, the promoter was predicted to be directly upstream of exon 1, including portions of exon 1 (Additional file 2: Figure S1). Our goal was to develop a low-cost assay that could be ported to large chemical libraries (> 100,000 compounds) that can be screened to identify and generate new chemical entities. For this reason, we selected a fluorescence-based reporter for the primary screening assay as gene activity can be rapidly imaged without the need for costly reagents. We used the immortalized C2C12 mouse myoblast cell line to generate stably transfected reporter cells containing an enhanced green fluorescent protein (EGFP) reporter driven by the hSSPN promoter (Fig. 2a). The hSSPN-EGFP myoblasts express reporter protein at increasing levels throughout differentiation revealing that the reporters are reflective of endogenous SSPN gene activity (Fig. 2b, c). Insulin transferrin selenium (ITS) was selected as the positive control for the screening assay as it broadly increases protein synthesis, resulting in an increase in SSPN. Treatment with 1% ITS induced a 1.4-fold increase in the hSSPN-EGFP reporter, mRNA, and protein levels after 48 h of treatment, showing that the reporter cells reflect the endogenous SSPN response to chemical stimulus (Additional file 6: Figure S4). Cells transfected with reporter plasmids lacking the SSPN promoter did not exhibit fluorescence reporter activity (data not shown). Transfection of the reporter construct into RAW264.7 macrophages (ATCC), a non-SSPN expressing cell line, indicated the cell-type specificity of the reporter (Additional file 7: Figure S5).

### Determining optimal assay conditions for high-throughput screening

Assay development is a challenging and laborious process that requires iterations of optimization. The obstacles that arise during assay development are confounded by the need to miniaturize assays and minimize the number of steps required, which both decrease handling time and directly increase throughput. To effectively screen large compound libraries, we miniaturized our assay to a 384-well microplate format, allowing for reduced reagent consumption and rapid data collection. To optimize assay conditions for high-throughput screening in a 384-well microplate format, we evaluated numerous parameters as listed in Additional file 8: Table S3. One limiting factor in high-throughput screens is the preparation of large quantities of cells. We therefore assessed multiple seeding densities ranging from 500 to 4000 cells per well and determined that seeding 500 cells per well followed by 3 days of incubation was sufficient for myoblasts to reach confluency before differentiation. This also reduced the amount of pre-screen culture required, making it an optimal condition for scaling up to meet high-throughput requirements. We tested the ability of the ITS positive control to increase reporter activity after 24 and 48 h and observed a detectable and significant change in reporter activity after 48 h of treatment (Additional file 6: Figure S4). Screening core facilities typically store small molecules in 100% DMSO, which can lead to cell toxicity from the DMSO vehicle alone. To assess DMSO toxicity in our cells, we dosed cells with 0.1–10% DMSO and observed a decrease in cell viability at 2% DMSO and higher after 48 h of treatment (data not shown). To ensure proper DMSO mixing to prevent regionally high concentrations of DMSO that can negatively impact cell viability, we tested several mixing methods and conditions. From these tests, we determined that the addition of 0.5 μl of 100% DMSO to an initial volume of 40 μl of media in each well, followed by 50 μl of additional media was sufficient to create a homogenous solution of DMSO, as detected by DMSO spiked with crystal violet dye (data not shown). Image analysis using a custom analysis module in MetaXpress software identifies myotubes based on specified dimensions and quantifies fluorescence pixel intensity of identified cells (Additional file 9: Figure S6). Imaging two regions of each well at × 10 magnification in low fluorescence media was sufficient for the custom analysis module to reliably detect a significant difference between vehicle and positive control treated cells.

### High-throughput screening on hSSPN-EGFP myotubes

**Fig. 3:**
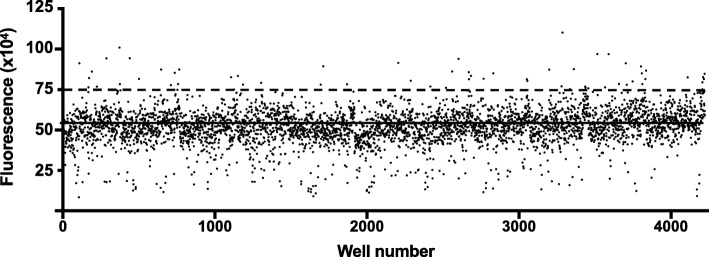
High-throughput screening of LOPAC, NIH, and Prestwick libraries on hSSPN-EGFP myotubes. hSSPN-EGFP cells differentiated for 2 days were used to screen 3200 molecules in the Library of Pharmacologically Active Compounds, NIHII, and Prestwick libraries (*n* = 1). The reporter cells were treated with 5.5 μM of compound and imaged 48 h later at day 4 of differentiation into myotubes. Two images per well were captured and analyzed using a MetaXpress custom analysis module to quantify fluorescence intensity. The solid line marking the average fluorescence of all data points and the dashed line marking the initial cutoff for hits (1.4-fold over vehicle)

To gain insight into the pathways involved in SSPN upregulation, we screened libraries of well-characterized FDA-approved compounds. Using the hSSPN-EGFP cells, we screened the Library of Pharmacologically Active Compounds (LOPAC), Prestwick Chemical (NPW), and NIHII small molecule libraries totaling 3200 small molecules (Fig. 3). All images were analyzed for cellular fluorescence intensity and compared with values from vehicle-treated cells. Robust strictly standardized mean difference (SSMD*) was used to classify the quality of each plate and the strength of each hit. All plates resulted in an SSMD* ≥ 1, indicating a good quality difference between vehicle and positive control treated cells (Table 1). Generally, SSMD* > 0.25 is the minimum to be considered a hit [40]. Based on the fold change detectable in cells treated with positive control, we set an initial hit cutoff of 1.4-fold fluorescence intensity over vehicle and eliminated images with debris or small molecules that auto-fluoresce, leading to a preliminary hit rate of 0.5% or 13 small molecules (Additional file 10: Table S4). Among hits, four were L-type calcium channel antagonists (felodipine, isradipine, lacidipine, nilvadipine) with felodipine appearing twice from the LOPAC and NPW libraries, indicating the robustness of the assay in reproducibly identifying hits. To confirm the hits, all 13 compounds were rescreened at 5.5 μM in 2 separate plates with *n* = 24 each plate (Additional file 11: Table S5). Of the 13 compounds, felodipine, GW5074, isoproterenol, isradipine, lacidipine, nandrolone, and nilvadipine resulted in an SSMD* > 0.25 (Table 2). In addition, GW5074, isoproterenol, and nandrolone are known to also affect intracellular calcium levels. These data strongly support that endogenous SSPN expression is regulated in a calcium-sensitive manner.

**Table 1:** Plate quality control using robust strictly standardized mean difference

Plate	Library	SSMD*
1	LOPAC1	1.2
2	LOPAC2	1.7
3	LOPAC3	1.3
4	LOPAC4	1.0
5	NPW1	1.7
6	NPW2	1.7
7	NPW3	1.0
8	NPW4	1.5
9	NIHII1	1.8
10	NIHII2	1.1
11	NIHII3	1.1

Robust strictly standardized mean difference (SSMD*) was used as a measurement of quality control. Each of the 11 plates resulted in an SSMD* ≥ 1, indicating a good quality effect size between vehicle and positive control treated cells

**Table 2:** Validated hits from hSSPN-EGFP screen reveal enrichment of calcium channel blockers

Compound	Description
Felodipine	L-type Ca2 + channel blocker
GW5074	cRaf1 kinase inhibitor
Isoproterenol	Sympathomimetic amine acting on β-adrenoceptors
Isradipine	L-type Ca2 + channel blocker
Lacidipine	L-type Ca2 + channel blocker
Nandrolone	Anabolic-androgenic steroid
Nilvadipine	L-type Ca2 + channel blocker

The screen resulted in 13 initial hits, which were further validated with the hSSPN-EGFP reporter with an *n* = 24. The 7 validated hits included an overrepresented number of L-type calcium channel blockers

### In vitro validation in wild-type and dystrophin-deficient muscle cells

**Fig. 4:**
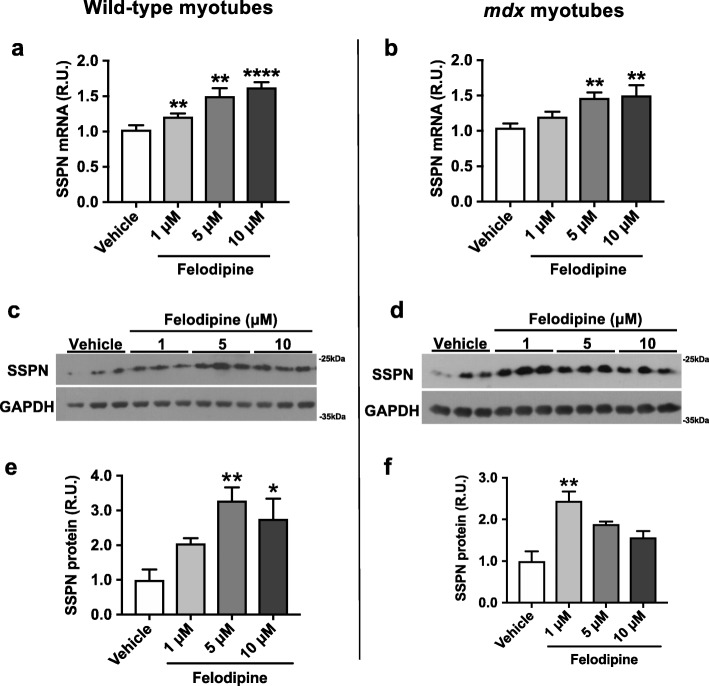
Compound identified in high-throughput screen increases sarcospan transcript and protein levels in both wild-type and dystrophin-deficient *mdx* myotubes. Felodipine increases sarcospan gene expression in **a** C2C12 wild-type and **b** *mdx* myotubes (*n* = 3–8) after 48 h of treatment. Gene expression was calculated using the ddCt method and normalized to β-actin with vehicle-treated cells serving as the calibrator sample. Using immunoblot analysis, total cell lysate from cells treated with felodipine was probed with anti-SSPN antibodies. GAPDH is shown as a loading control. Felodipine increases sarcospan protein levels in both **c** C2C12 wild-type and **d** and *mdx* myotubes (*n* = 3). **e**, **f** Quantification of SSPN protein levels are normalized to GAPDH. R.U., relative units. All data was obtained from murine myotubes treated for 48 h and assayed or harvested at day 4 of differentiation. *p < 0.05, **p < 0.01, ****p < 0.0001

To determine whether this increase in human SSPN promoter reporter activity translated to an increase in mouse SSPN transcript levels, we conducted follow-up assays using felodipine because of its effective dose-response profile in the hSSPN-EGFP cells and minimal effect on cell viability (Additional file 12: Figure S7; Additional file 13: Figure S8). We treated C2C12 myotubes with 1–10 μM of felodipine and observed that after 48 h of treatment, SSPN expression increased 1.5-fold over vehicle at the 10 μM dose (Fig. 4a). To assess the effect of felodipine in a dystrophin-deficient cell model, we used immortalized *mdx* myoblasts containing a nonsense mutation in exon 23 of the dystrophin gene. We treated *mdx* myotubes with 1–10 μM of felodipine and also observed a 1.5-fold increase in SSPN gene expression with the 10 μM treatment (Fig. 4b). Because increases in transcript levels do not necessarily correlate with changes in protein levels, we also assessed SSPN protein levels. Immunoblot analysis on wild-type C2C12 cells treated with 1–10 μM of felodipine revealed an increase in SSPN protein levels with 5 μM and 10 μM treatments (Fig. 4c, e). In *mdx* cells, treatment with 1 μM felodipine increased SSPN protein levels (Fig. 4d, f).

**Fig. 5:**
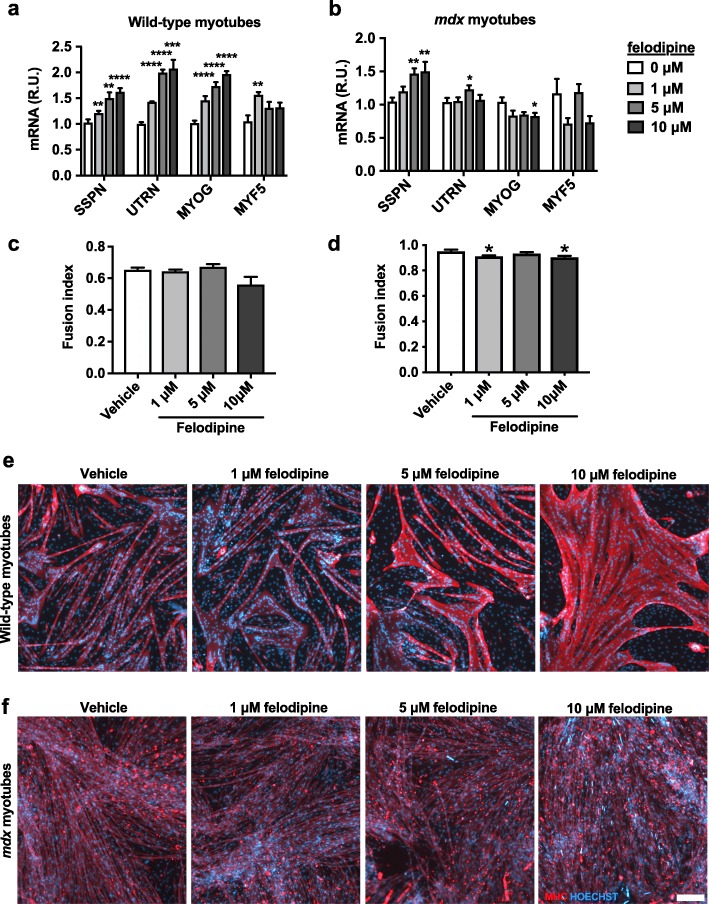
Felodipine enhances differentiation in wild-type, but not dystrophin-deficient myotubes. Felodipine increases utrophin and myogenin gene expression in **a** C2C12 wild-type myotubes, but not **b** *mdx* myotubes (*n* = 3–8) after 48 h of treatment (sarcospan gene expression data from the previous figure). **c**, **d** Felodipine does not increase fusion index (nuclei in myosin heavy chain positive area/total nuclei in field) in wild-type or *mdx* myotubes, **e**, **f** but does induce the formation of hypertrophic wild-type myotubes. Gene expression was calculated using the ddCt method and normalized to β-actin with vehicle-treated cells serving as the calibrator sample. UTRN, utrophin; MYOG, myogenin; MYF5, myogenic factor 5; MHC, myosin heavy chain. Scale bar, 200 μm. *p < 0.05, **p < 0.01, ***p < 0.001, ****p < 0.0001

To determine if felodipine was specifically increasing SSPN, we quantified gene expression of utrophin (UTRN), which increases with differentiation, and the myogenic markers myogenin (MYOG) and myogenic factor 5 (MYF5). In wild-type myotubes, felodipine increased SSPN, UTRN, and MYOG in a dose-dependent manner, indicating the treatment may accelerate differentiation (Fig. 5a). However, in *mdx* myotubes, only SSPN increased in a dose-dependent manner, suggesting that felodipine increases SSPN through pathways that are independent of differentiation (Fig. 5b). In both cell lines, MYF5 remained relatively stable as expected.

To determine if felodipine increases the rate of myoblast differentiation, we performed a fusion index assay on wild-type and *mdx* myotubes. Felodipine did not increase the number of cells fusing into myotubes in either cell line but did result in the formation of hypertrophic wild-type myotubes (Fig. 4c–f).

## Discussion

The heterogeneity of mutations and difficulty of delivery to muscle are major challenges in the development of urgently needed therapies to treat DMD. SSPN is able to reduce the pathology of muscular dystrophy in the DMD murine model by increasing membrane localization of the UGC and α7β1D-integrin adhesion complexes, effectively increasing laminin binding to compensate for the loss of dystrophin [25, 27–31]. Development of small molecule therapies that increase SSPN expression may lead to stand-alone or combinatorial therapies to treat DMD and other forms of muscular dystrophy caused by deficits in membrane proteins. Small molecule therapies are ideal due to their ability to bypass the limitations of delivery and immune responses seen with viral and cell-based methods.

In this study, we demonstrate the application of a cell-based reporter assay followed by in vitro hit-to-lead validation experiments that can be used to identify small molecule modulators of SSPN gene and protein expression. From our screen of 3200 small molecules using hSSPN-EGFP myotubes, we identified seven hits that increase reporter levels. We further validated the hit felodipine in wild-type and *mdx* myotubes and found that it increased endogenous SSPN mRNA and protein, showing that candidate drugs derived from the screen have the potential to activate both human and mouse SSPN. In wild-type myotubes, the increase in SSPN coincided with an increase in UTRN and MYOG, indicating that felodipine may be affecting differentiation and fusion of the myotubes. However, this effect was not observed in *mdx* myotubes. These differences in wild-type and *mdx* cell responses to treatment may be explained by findings that *mdx* mice show defective excitation-contraction coupling, altered calcium homeostasis, and altered expression of calcium handling proteins [43, 44].

We identified a class of dihydropyridine-derived calcium channel antagonists that increase SSPN reporter activity. Treatment of skeletal muscle cells with L-type calcium channel antagonists increases SSPN gene and protein expression, suggesting that endogenous SSPN gene activity is calcium sensitive. In cardiac and smooth muscle, L-type calcium channel antagonists interact directly with dihydropyridine receptors (DHPR) to reduce calcium movement through the channel and are used clinically to treat hypertension, regulate heart rate, and address chest pain. In skeletal muscle, DHPR are concentrated in the transverse (T)-tubules and are key regulators of excitation-contraction coupling. Upon depolarization along the T-tubules, DHPR activates the ryanodine receptor to release calcium from the sarcoplasmic reticulum to the cytosol, which facilitates the interaction between actin and myosin required for muscle contraction. DHPR forms a complex with an ATP release channel (pannexin 1), an ATP receptor (P2 purinoreceptor 2), caveolin-3, and dystrophin that is proposed to be involved in the excitation-transcription coupling, a calcium and ATP-sensitive regulation of gene expression [45]. In preclinical studies of dystrophic rodents, treatment with L-type calcium channel blocker nifedipine led to an increase in fiber diameter and muscle function and a decrease in serum creatine kinase levels [46, 47]. Multiple studies in human subjects show no improvement in disease outcome with long-term calcium channel antagonist treatment (for review, [48, 49]). However, calcium dysregulation is a hallmark feature in DMD and recent evidence points to a functional interplay between ion channels and adhesion complexes, indicating that calcium regulation is an important therapeutic target (for review, [48, 49]).

In conclusion, we developed and validated a cell-based reporter assay that effectively identifies compounds that enhance SSPN gene and protein expression in wild-type and dystrophin-deficient myotubes. Future studies will be focused on porting our validated assay system to a larger chemical space to identify and develop new chemical entities for the treatment of DMD.

## Conclusions

- Gene expression analysis of individual components of the DGC and UGC revealed an exponential increase in mRNA levels beginning at day 3 of differentiation. During differentiation, SSPN, UTRN, ITGA7, and ITGB1 increase to similar levels (tenfold over myoblast levels). DAG, SCGA, and SCGB increase 50- to 100-fold, while DMD increases up to 1000-fold over myoblast levels.
- Screening of FDA-approved drug libraries on hSSPN-EGFP murine myotubes revealed enrichment in L-type calcium channel antagonists, indicating a new potential role in calcium regulation of SSPN expression.
- Compounds identified in the screen increase hSSPN-EGFP activity as well as SSPN mRNA and protein levels in wild-type and dystrophin-deficient *mdx myotubes*, demonstrating the ability of the assay to identify relevant compounds that are active in both human, mouse, wild-type, and *mdx* muscle cells.
- In summary, we developed a high-throughput assay using C2C12 myotubes stably transfected with a hSSPN-EGFP reporter that is fully optimized for a 384-well microplate format with minimal reagents and handling and can thus be used for large-scale screens.

## Supplementary information

**Additional file 1: Table S1.** Primers used for gene expression analysis. Primers optimized by standard curve method using cDNA corresponding to 75 ng RNA, diluted 2-fold. AE: amplification efficiency, calculated using the eq. AE = [10(− 1/slope)]/ – 1. SSPN, sarcospan; DMD, dystrophin; UTRN, utrophin; DAG, dystroglycan, SCGA, α-sarcoglycan; SCGB, β-sarcoglycan; ITGA7, α7 integrin; ITGB1, β1D integrin; MYOG, myogenin; MYF5, myogenic factor 5; ACTB, β-actin; GADPH, glyceraldehyde 3-phosphate dehydrogenase.

**Additional file 2: Figure S1.** Predicted human SSPN promoter region determined using UCSC genome browser. H2K4me3 marks, DNase hypersensitivity regions, and ChIP-seq data reveal predicted transcription factor binding sites in human skeletal muscle cultures. These analyses indicate that the SSPN promoter includes region upstream of exon 1 and within exon 1. Shown is skeletal muscle and cardiac-specific transcript variant 1 (NM_005086.4) of the human SSPN gene (NG_012011.2) in UCSC Genome browser human Feb. 2009 (GRCh37/hg19) assembly. Location shown: chr12:26,342,405-26,392,014.

**Additional file 3: Table S2.** Primers used for reporter construct cloning and sequencing. Cloning optimized for human sarcospan (SSPN) gene region and pmEGFP-1 plasmid (EGFP).

**Additional file 4: Figure S2.** C2C12 myoblasts undergoing differentiation and fusion into myotubes. Confluent C2C12 myoblasts (day 0, D0) were switched from proliferation to differentiation media and imaged daily using phase contrast microscopy for 7 days (D1 to D7). Scale bar = 200 μm.

**Additional file 5: Figure S3.** Summary of gene expression of myofiber membrane adhesion complex members during C2C12 differentiation. Expression of individual genes encoding protein components of the three major adhesion complexes (DGC, UGC, and α7β1D-integrin complex) were investigated, including: (a) SSPN, sarcospan; (b) DMD, dystrophin; (c) UTRN, utrophin; (d) DAG, dystroglycan, (e) SCGA, α-sarcoglycan; (f) SCGB, β-sarcoglycan; (g) ITGA7, α7 integrin; and (h) ITGB1, β1D integrin. Gene expression was calculated using the ddCt method and normalized to β-actin with day 0 (myoblast) values serving as the calibrator sample (*n* = 3). R.U., relative units.

**Additional file 6: Figure S4.** Sarcospan reporter, gene, and protein levels increase similarly after treatment with positive control. Treatment with positive control, 1% insulin transferrin selenium (ITS), increased (a) reporter levels of hSSPN-EGFP reporter cells (*n* = 10–21), (b) SSPN mRNA in wild-type C2C12 cells, and (c-d) SSPN protein in wild-type C2C12 cells. Images of the hSSPN-EGFP cells were analyzed using a MetaXpress custom analysis module. Gene expression was calculated using the ddCt method and normalized to β-actin with vehicle control values serving as the calibrator sample. For immunoblot analysis, total cell lysate was probed with anti-SSPN antibody. GAPDH is shown as a loading control. Quantification for immunoblot shown in panel (d). All cells were treated at day 2 of differentiation and assayed at day 4 of differentiation. Data reported as fold change over vehicle-treated cells. *** *p* < 0.001, **** *p* < 0.0001.

**Additional file 7: Figure S5.** hSSPN-EGFP construct is expressed in a cell-type specific manner. The hSSPN-EGFP plasmid was transfected into C2C12 murine myoblasts or RAW264.7 murine macrophages. At 48 h and 7 days post-transfection EGFP was detected in the myoblasts, but not the macrophages. Scale bar = 100 μm.

**Additional file 8: Table S3.** Assay parameters optimized for high-throughput screening of hSSPN-EGFP myotubes. High-throughput assay conditions were optimized for a 384-well microplate format screen on hSSPN-EGFP reporter myotubes treated with a concentrated stock solution of 1 mM small molecule in 100% DMSO. Assay parameters optimized for C2C12 myotube screening were developed in the Molecular Screening Shared Resource facility using specialized, automated equipment (see Methods).

**Additional file 9: Figure S6.** High-content image analysis workflow. The MetaXpress custom analysis module processed images from high-content imaging by transforming the input image to remove background (Top Hat Analysis), identifying cells by size and intensity above local background (Adaptive Threshold), excluding debris by minimum area (Filter Mask), and quantifying fluorescence intensity of each resulting image. Scale bar, 220 μm.

**Additional file 10: Table S4.** Initial hits from high-throughput screening of hSSPN-EGFP myotubes. The screen of 3200 compounds resulted in 13 initial hits, which included an overrepresented number of L-type calcium channel blockers (felodipine, isradipine, lacidipine, nifedipine, and nilvadipine) and one hit (felodipine) which appeared as a hit twice from two independent libraries. Robust strictly standardized mean difference (SSMD*) was used to classify the strength of each hit. SSMD* > 0.25 and 1.4-fold change over vehicle was the minimum to be considered an initial hit. R.U., relative units normalized to vehicle control.

**Additional file 11: Table S5.** Validation of hits from screen on hSSPN-EGFP myotubes. Initial hits from the screen were retested on hSSPN-EGFP myotubes at 5.5 μM in 2 plates (*n* = 24 per plate). R.U., relative units normalized to vehicle control. *SSMD, robust strictly standardized mean difference.

**Additional file 12: Figure S7.** Titration of screen hits on hSSPN-EGFP myotubes. hSSPN-EGFP myotubes were treated with 1 nM-20 μM of (a) felodipine of (b) nilvadipine for 48 h and imaged at day 4 of differentiation using a high-content imager (*n* = 12). Images were analyzed using a MetaXpress custom module to calculate fluorescence over vehicle (R.U., relative units).

**Additional file 13: Figure S8.** Effect of felodipine and nilvadipine on cell viability of C2C12 wild-type and H2K *mdx* myotubes. C2C12 wild-type and H2K *mdx* myotubes were treated with 1.25–40 μM of felodipine for 48 h and assayed at day 4 of differentiation using an ATP-based cell viability assay.

## Data Availability

Not applicable.

## Abbreviations

DAG: Dystroglycan
DGC: Dystrophin-glycoprotein complex
DMD: Duchenne muscular dystrophy/dystrophin
DMSO: Dimethyl sulfoxide
ECM: Extracellular matrix
EGFP: Enhanced green fluorescent protein
hSSPN: Human sarcospan
HTS: High-throughput screening
ITGA7: α7 integrin
ITGB1: β1D integrin
ITS: Insulin transferrin selenium
MTA: Methylthioadenosine
MYF5: Myogenic factor 5
MYOG: Myogenin
SCGB: β-Sarcoglycan
SGCA: α-Sarcoglycan
SSPN: Sarcospan
UGC: Utrophin-glycoprotein complex
UTRN: Utrophin
