# Supplementary material for: Development of a high-throughput screen to identify small molecule enhancers of sarcospan for the treatment of Duchenne muscular dystrophy
Source: Skelet Muscle. 2019 Dec 12;9:32. doi: 10.1186/s13395-019-0218-x (PMC6907331; doi:10.1186/s13395-019-0218-x)
Supplement: Supplementary file 1 — Additional file 1: Table S1. Primers used for gene expression analysis. Primers optimized by standard curve method using cDNA corresponding to 75 ng RNA, diluted 2-fold. AE: amplification efficiency, calculated using the eq. AE = [10(− 1/slope)]/ – 1. SSPN, sarcospan; DMD, dystrophin; UTRN, utrophin; DAG, dystroglycan, SCGA, α-sarcoglycan; SCGB, β-sarcoglycan; ITGA7, α7 integrin; ITGB1, β1D integrin; MYOG, myogenin; MYF5, myogenic factor 5; ACTB, β-actin; GADPH, glyceraldehyde 3-phosphate dehydrogenase. [file 13395_2019_218_MOESM1_ESM.pdf]

PRIMER	SEQUENCE (5' → 3')	LOCATION	AMPLICON LENGTH	AE
UTRN F	GTATGGGGACCTTGAAGCCAG	exons 1-2	125BP	118%
UTRN R	ATCGAGCGTTTATCCATTTGGT			
DMD F	GGAAAGCAACACATAGACAACCT	exons 3-4	65BP	111%
DMD R	GGGCATGAACTCTTGTAGATCC			
ITGA7 F	GATCGTCCGAGCCAACATCACA	exons 23-24	165BP	115%
ITGA7 R	CTAACAGCCCAGCCAGCACT			
ITGB1 F	ATGCCAAATCTTGCGGAGAAT	exons 3-4	209BP	105%
ITGB1 R	TTTGCTGCGATTGGTGACATT			
DAG1 F	CAGACGGTACGGCTGTTGTC	exons 3-4	126BP	112%
DAG1 R	AGTGTAGCCAAGACGGTAAGG			
SGCA F	GCAGCAGTAACTTGGATACCTC	exons 2-3	113BP	117%
SGCA R	AAAGGATGCACAAACACACGA			
SGCB F	AGCACAACAGCAATTTCAAAGC	exon 2	112BP	100%
SGCB R	AGGAGGACGATCACGCAGAT			
SSPN F	TGCTAGTCAGAGATACTCCGTTC	exons 1-2	103BP	94%
SSPN R	GTCCTCTCGTCAACTTGGTATG			
MYOG F	GAGATCCTGCGCAGCGCCAT	exon 1	97BP	107%
MYOG R	CCCCGCCTCTGTAGCGGAGA			
MYF5 F	AAGGCTCCTGTATCCCCTCAC	exon 1	249BP	117%
MYF5 R	TGACCTTCTTCAGGCGTCTAC			
ACTB F	TCCTGACCCTGAAGTACCCCAT	exons 1-2	131BP	104%
ACTB R	CTCGGTGAGCAGCACAGGGT			
GAPDH F	CAACTTTGGCATTGTGGAAGG	exons 4-5	135BP	92%
GAPDH R	GTGGATGCAGGGATGATGTT			
