# Supplementary figures and images for: Development of a high-throughput screen to identify small molecule enhancers of sarcospan for the treatment of Duchenne muscular dystrophy
Source: Skelet Muscle. 2019 Dec 12;9:32. doi: 10.1186/s13395-019-0218-x (PMC6907331; doi:10.1186/s13395-019-0218-x)

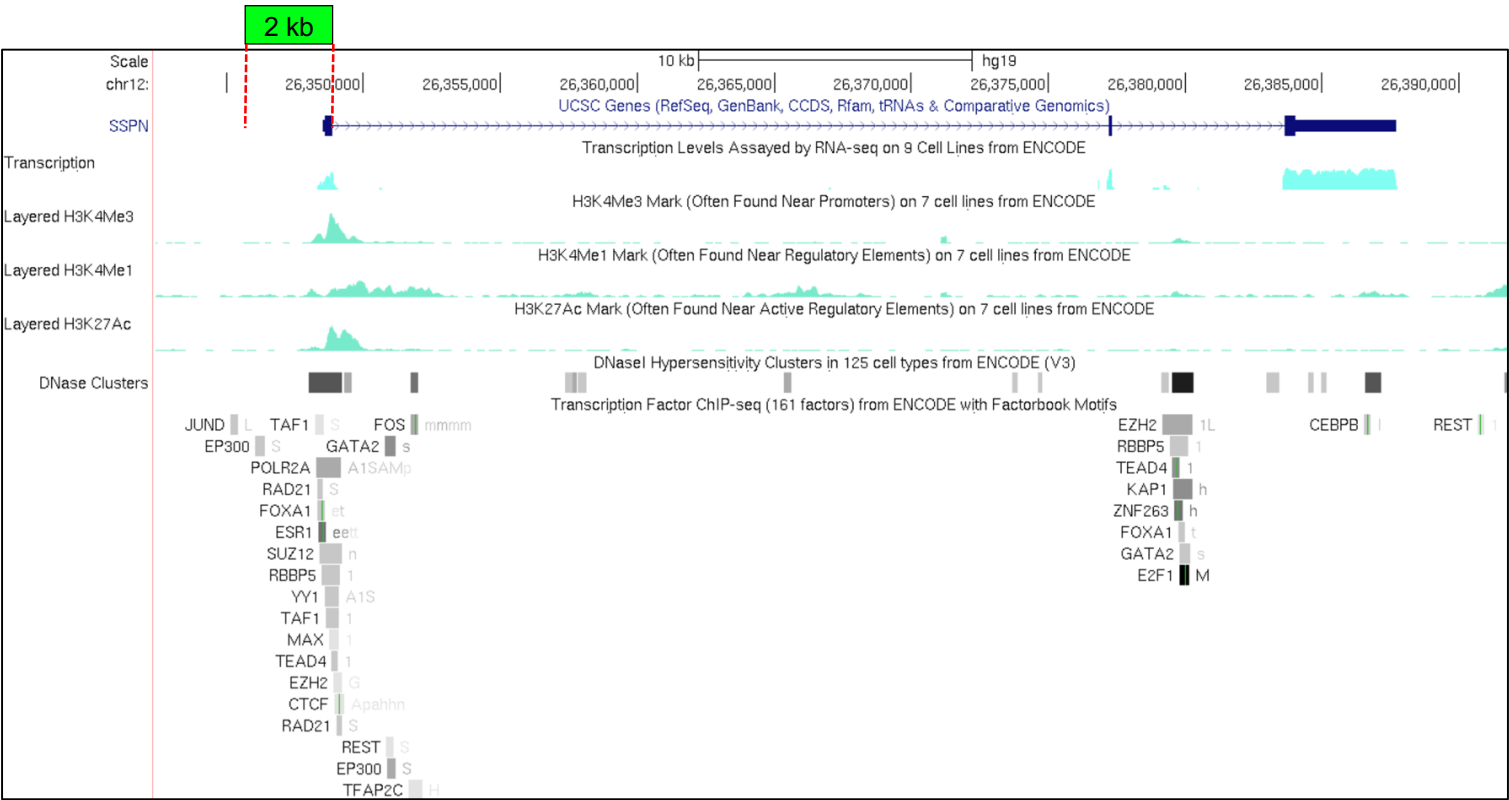

Supplement: Supplementary file 2 — Additional file 2: Figure S1. Predicted human SSPN promoter region determined using UCSC genome browser. H2K4me3 marks, DNase hypersensitivity regions, and ChIP-seq data reveal predicted transcription factor binding sites in human skeletal muscle cultures. These analyses indicate that the SSPN promoter includes region upstream of exon 1 and within exon 1. Shown is skeletal muscle and cardiac-specific transcript variant 1 (NM_005086.4) of the human SSPN gene (NG_012011.2) in UCSC Genome browser human Feb. 2009 (GRCh37/hg19) assembly. Location shown: chr12:26,342,405-26,392,014. [file 13395_2019_218_MOESM2_ESM.pdf]

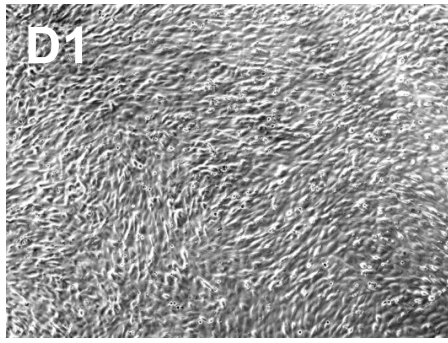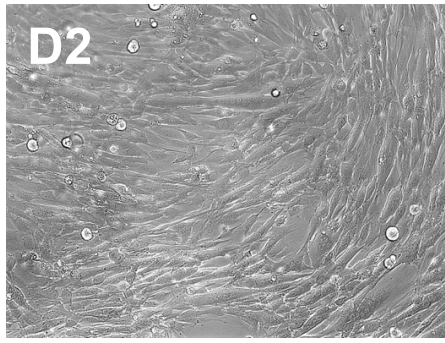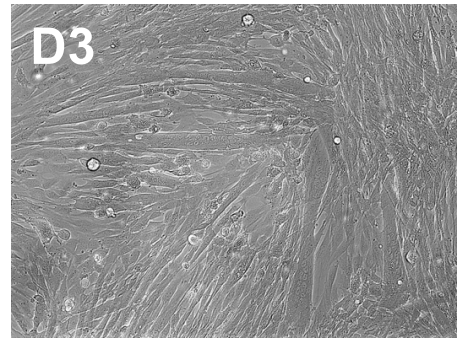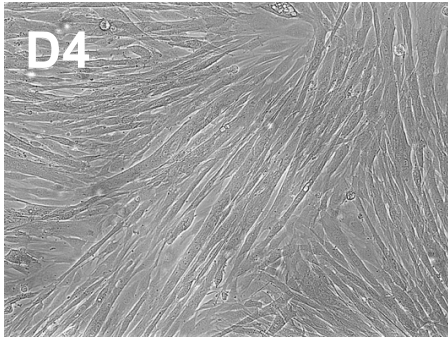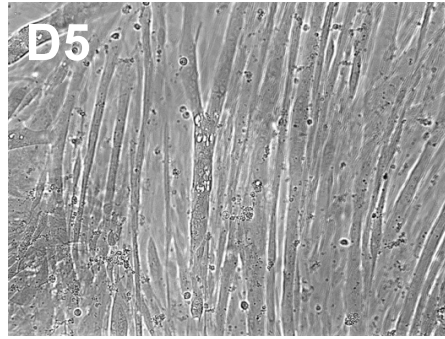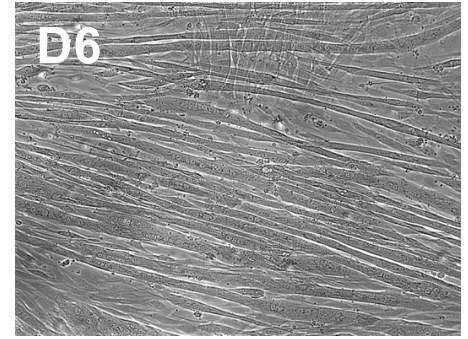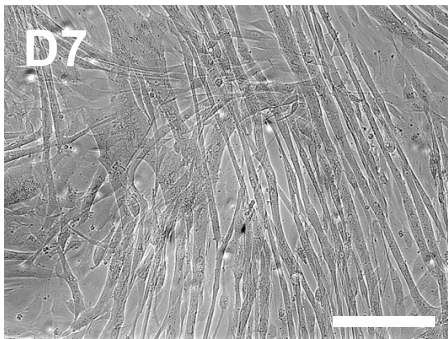

Supplement: Supplementary file 4 — Additional file 4: Figure S2. C2C12 myoblasts undergoing differentiation and fusion into myotubes. Confluent C2C12 myoblasts (day 0, D0) were switched from proliferation to differentiation media and imaged daily using phase contrast microscopy for 7 days (D1 to D7). Scale bar = 200 μm. [file 13395_2019_218_MOESM4_ESM.pdf]

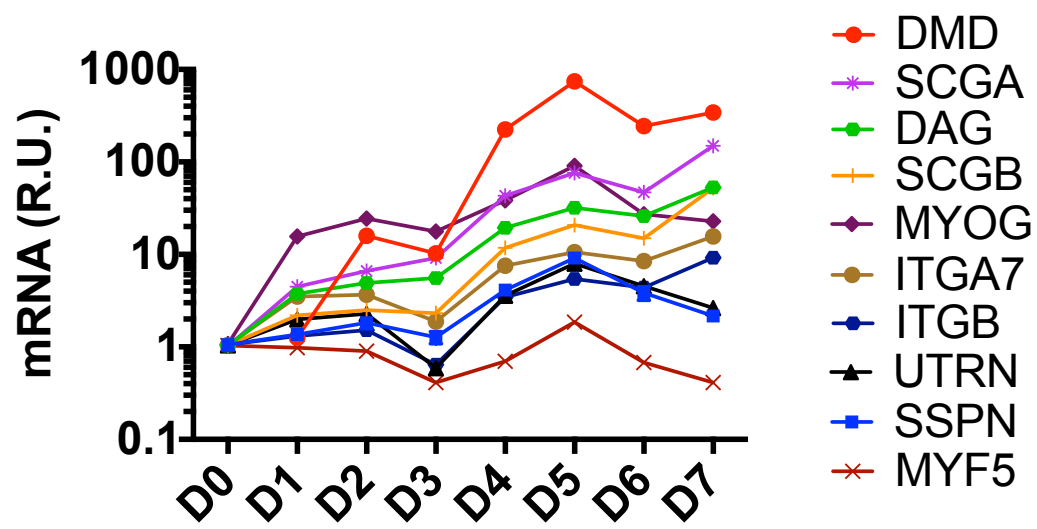

Supplement: Supplementary file 5 — Additional file 5: Figure S3. Summary of gene expression of myofiber membrane adhesion complex members during C2C12 differentiation. Expression of individual genes encoding protein components of the three major adhesion complexes (DGC, UGC, and α7β1D-integrin complex) were investigated, including: (a) SSPN, sarcospan; (b) DMD, dystrophin; (c) UTRN, utrophin; (d) DAG, dystroglycan, (e) SCGA, α-sarcoglycan; (f) SCGB, β-sarcoglycan; (g) ITGA7, α7 integrin; and (h) ITGB1, β1D integrin. Gene expression was calculated using the ddCt method and normalized to β-actin with day 0 (myoblast) values serving as the calibrator sample (n = 3). R.U., relative units. [file 13395_2019_218_MOESM5_ESM.pdf]

**a**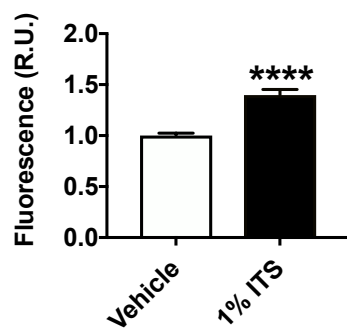**b**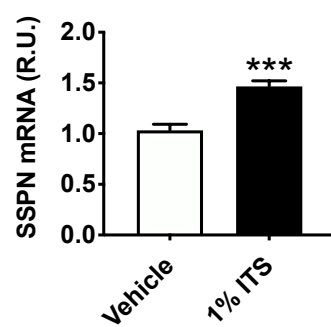**c**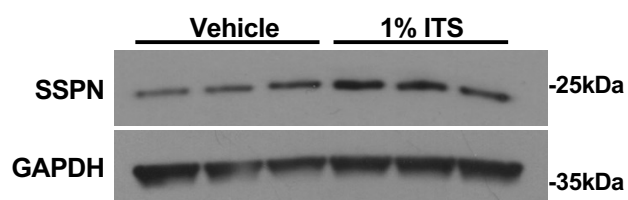**d**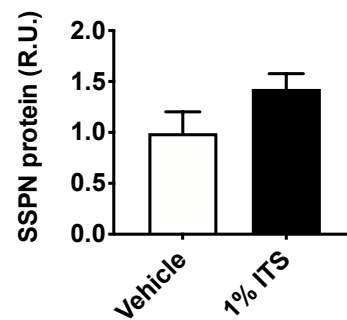

Supplement: Supplementary file 6 — Additional file 6: Figure S4. Sarcospan reporter, gene, and protein levels increase similarly after treatment with positive control. Treatment with positive control, 1% insulin transferrin selenium (ITS), increased (a) reporter levels of hSSPN-EGFP reporter cells (n = 10–21), (b) SSPN mRNA in wild-type C2C12 cells, and (c-d) SSPN protein in wild-type C2C12 cells. Images of the hSSPN-EGFP cells were analyzed using a MetaXpress custom analysis module. Gene expression was calculated using the ddCt method and normalized to β-actin with vehicle control values serving as the calibrator sample. For immunoblot analysis, total cell lysate was probed with anti-SSPN antibody. GAPDH is shown as a loading control. Quantification for immunoblot shown in panel (d). All cells were treated at day 2 of differentiation and assayed at day 4 of differentiation. Data reported as fold change over vehicle-treated cells. *** p < 0.001, **** p < 0.0001. [file 13395_2019_218_MOESM6_ESM.pdf]

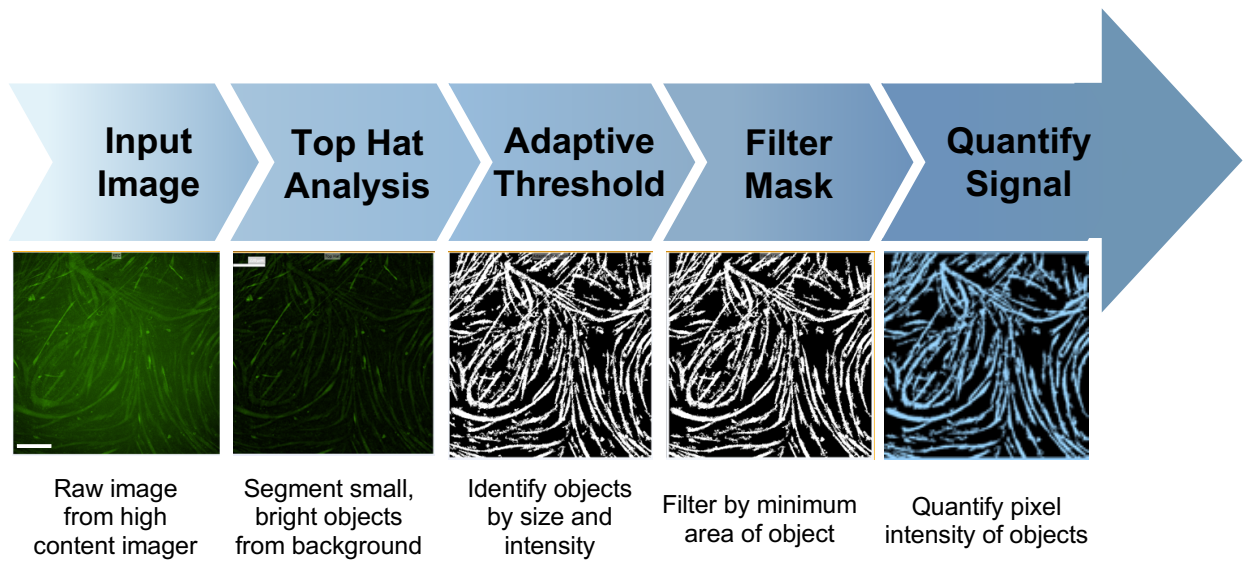

Supplement: Supplementary file 9 — Additional file 9: Figure S6. High-content image analysis workflow. The MetaXpress custom analysis module processed images from high-content imaging by transforming the input image to remove background (Top Hat Analysis), identifying cells by size and intensity above local background (Adaptive Threshold), excluding debris by minimum area (Filter Mask), and quantifying fluorescence intensity of each resulting image. Scale bar, 220 μm. [file 13395_2019_218_MOESM9_ESM.pdf]

**a**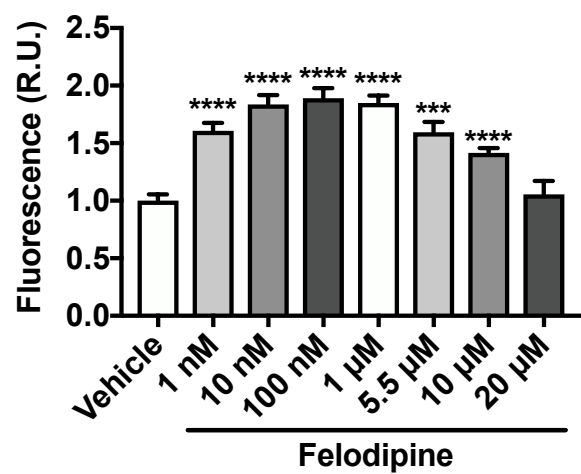**b**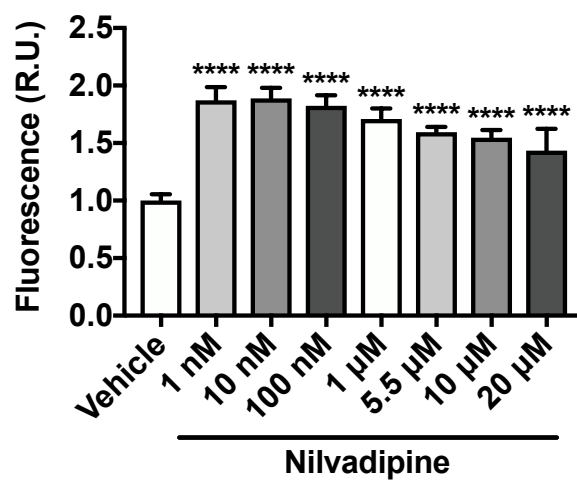

Supplement: Supplementary file 12 — Additional file 12: Figure S7. Titration of screen hits on hSSPN-EGFP myotubes. hSSPN-EGFP myotubes were treated with 1 nM-20 μM of (a) felodipine of (b) nilvadipine for 48 h and imaged at day 4 of differentiation using a high-content imager (n = 12). Images were analyzed using a MetaXpress custom module to calculate fluorescence over vehicle (R.U., relative units). [file 13395_2019_218_MOESM12_ESM.pdf]

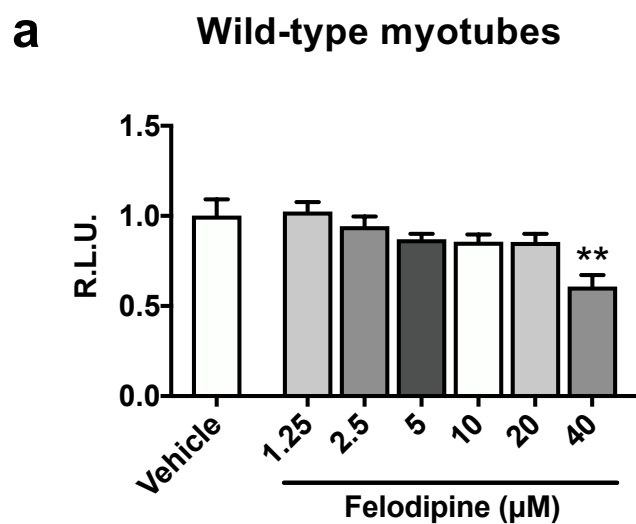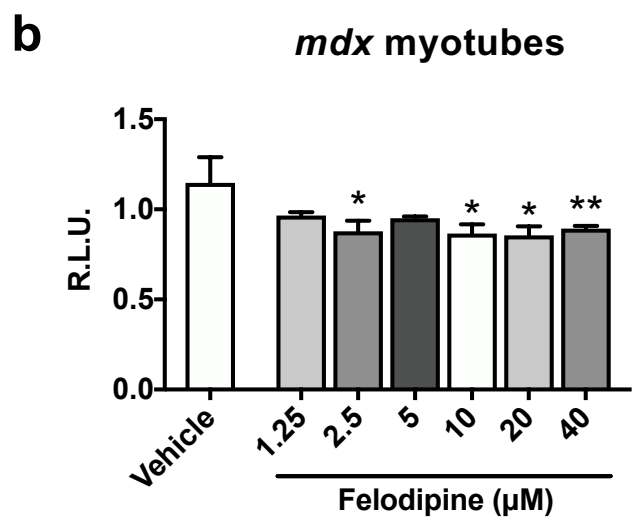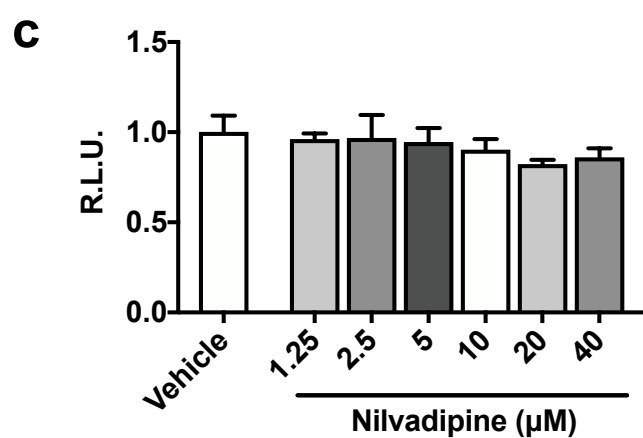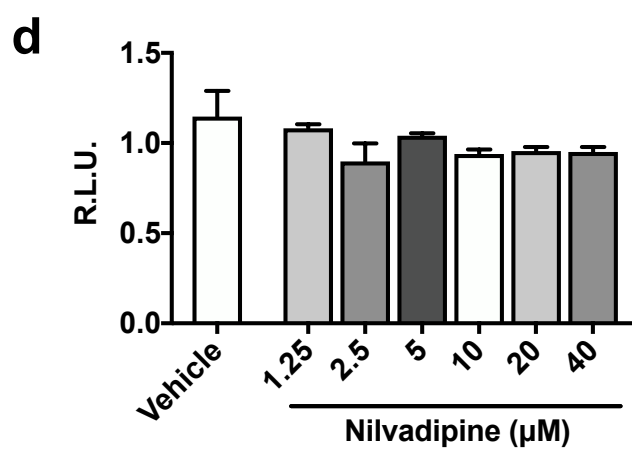

Supplement: Supplementary file 13 — Additional file 13: Figure S8. Effect of felodipine and nilvadipine on cell viability of C2C12 wild-type and H2K mdx myotubes. C2C12 wild-type and H2K mdx myotubes were treated with 1.25–40 μM of felodipine for 48 h and assayed at day 4 of differentiation using an ATP-based cell viability assay. [file 13395_2019_218_MOESM13_ESM.pdf]
