# Supplementary material for: Development of a high-throughput screen to identify small molecule enhancers of sarcospan for the treatment of Duchenne muscular dystrophy
Source: Skelet Muscle. 2019 Dec 12;9:32. doi: 10.1186/s13395-019-0218-x (PMC6907331; doi:10.1186/s13395-019-0218-x)
Supplement: Supplementary file 3 — Additional file 3: Table S2. Primers used for reporter construct cloning and sequencing. Cloning optimized for human sarcospan (SSPN) gene region and pmEGFP-1 plasmid (EGFP). [file 13395_2019_218_MOESM3_ESM.pdf]

PRIMER	SEQUENCE (5' → 3')	LOCATION
EGFP construct forward primer	GTGTAGATCTCAGGTGGGTGTCCTGGTATAA	2kb upstream of hSSPN TSS
EGFP construct reverse primer	GTGTAAGCTTCTCCTCCCCGCACTCCTT	Exon 1 of hSSPN
EGFP sequencing forward primer 1	ATAACCGTATTACCGCCATGCATTA	25bp upstream of hSSPN promoter
EGFP sequencing forward primer 2	CTCTAAGTGCTACTGAGTAGAGGTA	600bp within hSSPN promoter
EGFP sequencing forward primer 3	CAGCCACTTGGAGACTGAGGAGAGA	1200bp within hSSPN promoter
