# Supplementary material for: Development of a high-throughput screen to identify small molecule enhancers of sarcospan for the treatment of Duchenne muscular dystrophy
Source: Skelet Muscle. 2019 Dec 12;9:32. doi: 10.1186/s13395-019-0218-x (PMC6907331; doi:10.1186/s13395-019-0218-x)
Supplement: Supplementary file 7 — Additional file 7: Figure S5. hSSPN-EGFP construct is expressed in a cell-type specific manner. The hSSPN-EGFP plasmid was transfected into C2C12 murine myoblasts or RAW264.7 murine macrophages. At 48 h and 7 days post-transfection EGFP was detected in the myoblasts, but not the macrophages. Scale bar = 100 μm. [file 13395_2019_218_MOESM7_ESM.pdf]

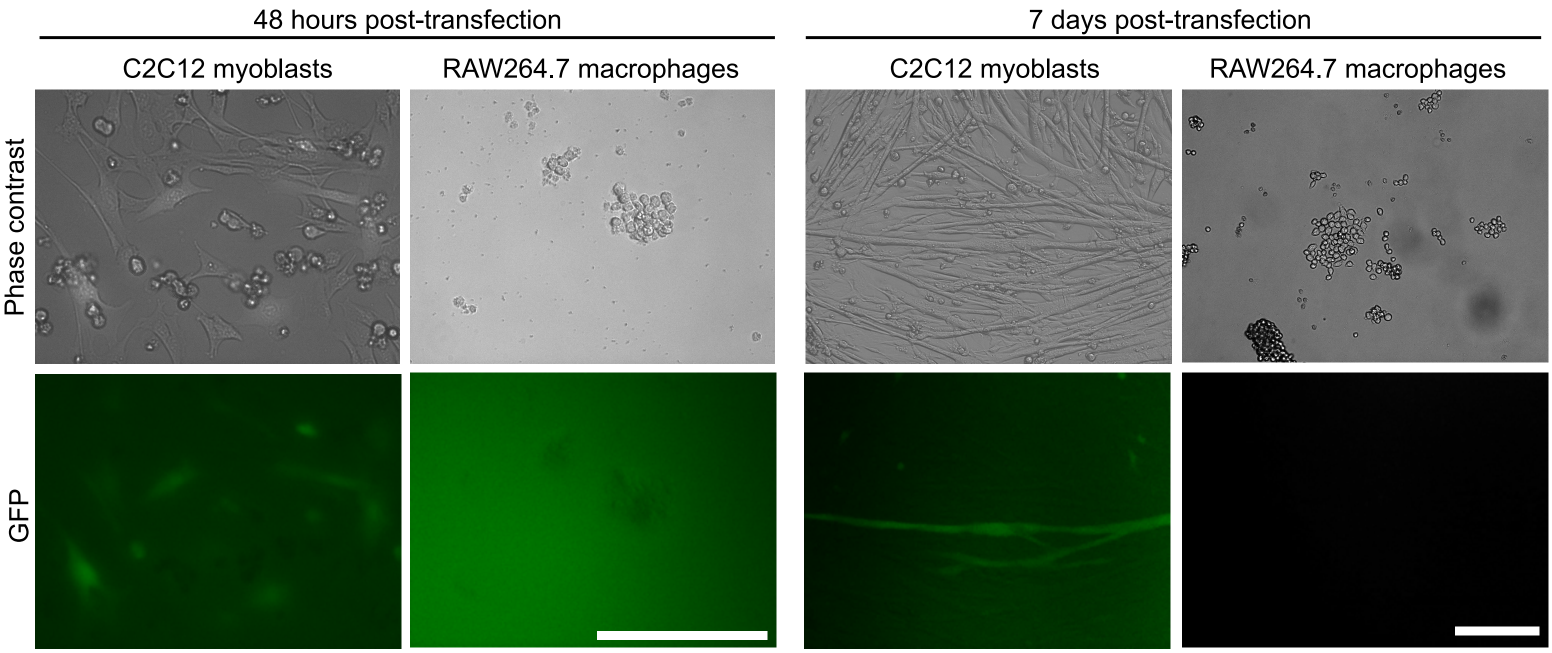
48 hours post-transfection
7 days post-transfection
C2C12 myoblasts
RAW264.7 macrophages
C2C12 myoblasts
RAW264.7 macrophages
Phase contrast
GFP
