# Supplementary material for: Development of a high-throughput screen to identify small molecule enhancers of sarcospan for the treatment of Duchenne muscular dystrophy
Source: Skelet Muscle. 2019 Dec 12;9:32. doi: 10.1186/s13395-019-0218-x (PMC6907331; doi:10.1186/s13395-019-0218-x)
Supplement: Supplementary file 8 — Additional file 8: Table S3. Assay parameters optimized for high-throughput screening of hSSPN-EGFP myotubes. High-throughput assay conditions were optimized for a 384-well microplate format screen on hSSPN-EGFP reporter myotubes treated with a concentrated stock solution of 1 mM small molecule in 100% DMSO. Assay parameters optimized for C2C12 myotube screening were developed in the Molecular Screening Shared Resource facility using specialized, automated equipment (see Methods). [file 13395_2019_218_MOESM8_ESM.pdf]

Assay component	Optimized condition
Plate format	384-well black clear-bottom microplates
Seeding density	500 cells/well
Differentiation protocol	Serum deprivation at ~90% cell confluency (3 days post-seeding)
Treatment period	48 hours beginning at day 2 of differentiation
Vehicle/DMSO toxicity	Viability unaffected at <2% DMSO
Mixing	Mix DMSO by media addition to treated wells
Positive control	1% insulin transferrin selenium for 48 hours
Treatment dose	5.5 μM compound
Final DMSO conc.	0.55% DMSO
Imaging conditions	Fluorobrite low fluorescence media, 10X, 2 tile capture (40% well coverage)
Analysis	Custom analysis module to detect fluorescence intensity
