# Supplementary material for: Development of a high-throughput screen to identify small molecule enhancers of sarcospan for the treatment of Duchenne muscular dystrophy
Source: Skelet Muscle. 2019 Dec 12;9:32. doi: 10.1186/s13395-019-0218-x (PMC6907331; doi:10.1186/s13395-019-0218-x)
Supplement: Supplementary file 10 — Additional file 10: Table S4. Initial hits from high-throughput screening of hSSPN-EGFP myotubes. The screen of 3200 compounds resulted in 13 initial hits, which included an overrepresented number of L-type calcium channel blockers (felodipine, isradipine, lacidipine, nifedipine, and nilvadipine) and one hit (felodipine) which appeared as a hit twice from two independent libraries. Robust strictly standardized mean difference (SSMD*) was used to classify the strength of each hit. SSMD* > 0.25 and 1.4-fold change over vehicle was the minimum to be considered an initial hit. R.U., relative units normalized to vehicle control. [file 13395_2019_218_MOESM10_ESM.pdf]

Compound	Fluorescence (R.U.)	SSMD*	Description
Aceclidine	1.4	3.4	agonist of muscarinic receptors
Acyclovir	2.0	3.4	Herpesvirus DNA polymerase inhibitor
Alloxazine	1.9	3.2	Selective A2b adenosine receptor antagonist
Carbadox	1.4	3.6	antibacterial
Felodipine	1.8	3.2	L-type Ca^{2+} channel blocker
Felodipine	1.9	5.8	L-type Ca^{2+} channel blocker
GW5074	1.6	2.6	cRaf1 kinase inhibitor
Isradipine	1.6	2.3	L-type Ca^{2+} channel blocker
Lacidipine	1.4	3.8	L-type Ca^{2+} channel blocker
Nafadotride	1.5	2	dopamine antagonist
Nandrolone	2.7	5.2	anabolic-androgenic steroid
Nifedipine	1.4	1.9	L-type Ca^{2+} channel blocker
Nilvadipine	1.8	2.3	L-type Ca^{2+} channel blocker
Isoproterenol	1.7	3.1	Sympathomimetic amine acting on β-adrenoceptors
