# Supplementary material for: Development of a high-throughput screen to identify small molecule enhancers of sarcospan for the treatment of Duchenne muscular dystrophy
Source: Skelet Muscle. 2019 Dec 12;9:32. doi: 10.1186/s13395-019-0218-x (PMC6907331; doi:10.1186/s13395-019-0218-x)
Supplement: Supplementary file 11 — Additional file 11: Table S5. Validation of hits from screen on hSSPN-EGFP myotubes. Initial hits from the screen were retested on hSSPN-EGFP myotubes at 5.5 μM in 2 plates (n = 24 per plate). R.U., relative units normalized to vehicle control. *SSMD, robust strictly standardized mean difference. [file 13395_2019_218_MOESM11_ESM.pdf]

	hSSPN-EGFP			
	Plate 1		Plate 2	
Compound	R.U.	SSMD*	R.U.	SSMD*
Aceclidine	1.1	-0.07	1.1	0.25
Acyclovir	0.9	-0.37	1.0	0.19
Alloxazine	1.0	-0.03	1.1	0.21
Carbadox	1.1	0.21	1.1	0.43
Felodipine	1.4	1.75	1.4	1.56
GW5074	1.2	0.67	1.2	0.78
Isoproterenol	1.1	0.26	1.1	0.26
Isradipine	1.5	1.79	1.4	1.50
Lacidipine	1.3	0.69	1.2	1.02
Nafadotride	1.1	-0.09	1.0	-0.01
Nandrolone	1.2	0.44	1.1	0.41
Nifedipine	1.3	0.41	1.0	0.22
Nilvadipine	1.6	1.08	1.5	0.97
